# Comparison of Mortality and Comorbidity Rates Between Holocaust Survivors and Individuals in the General Population in Israel

**DOI:** 10.1001/jamanetworkopen.2018.6643

**Published:** 2019-01-04

**Authors:** Naama Fund, Nachman Ash, Avi Porath, Varda Shalev, Gideon Koren

**Affiliations:** 1 Maccabi Kahn Institute for Research and Innovation, Maccabi Healthcare Services, Tel Aviv, Israel; 2 Faculty of Medicine, Ariel University, Ariel, Israel; 3 Department of Epidemiology, Ben Gurion University of the Negev, Beersheba, Israel; 4 Department of Pediatrics, Tel Aviv University, Tel Aviv, Israel; 5 Department of Pediatrics, Western University, London, Ontario, Canada

## Abstract

**Importance:**

Previous studies have suggested that Holocaust survivors may experience different chronic comorbidities more often than the general population. However, the mortality hazard among these individuals has not been addressed.

**Objective:**

To assess the overall mortality rate and comorbidities of a cohort of Holocaust survivors compared with an age-matched control group.

**Design, Setting, and Participants:**

This cross-sectional study included all Holocaust survivors insured by Maccabi Healthcare Services in Israel who were born between 1911 and 1945 in Europe and control individuals born in Israel during the same years and insured by the same service. Data were collected from January 1, 1998, through December 31, 2017.

**Outcomes and Measures:**

Rates of morbidities and mortality rates adjusted for sex, socioeconomic status, and body mass index using logistic regression, Cox regression, and Kaplan-Meier analysis.

**Results:**

The 38 597 Holocaust survivors included 22 627 women (58.6%) and had a mean (SD) age of 81.7 (5.4) years, and the 34 931 individuals in the control group included 18 615 women (53.3%) and had a mean (SD) age of 77.7 (5.3) years. The Holocaust survivors had higher rates than control individuals of reported hypertension (32 038 [83.0%] vs 23 285 [66.7]), obesity (12 838 [33.3%] vs 9254 [26.5]), chronic kidney disease (11 929 [30.9%] vs 6927 [19.8]), cancer (11 369 [29.5%] vs 9721 [27.8]), dementia (6389 [16.6%] vs 3355 [9.6]), ischemic heart disease, nonmyocardial infarction (5729 [14.8%] vs 4135 [11.8]), myocardial infarction (3641 [9.4%] vs 2723 [7.8]), and osteoporotic fractures among women (6429 [28.4%] vs 4120 [22.1]). In contrast, the overall mortality rate was lower among Holocaust survivors (25.3%) compared with the control group (41.1%). After adjustment for confounders, mean age at death was significantly higher in the survivor group compared with the control group.

**Conclusions and Relevance:**

The findings showed higher rates of comorbidities and lower mortality among Holocaust survivors, which may be associated with a combination of improved health literacy and unique resilience characteristics among Holocaust survivors. More research is needed to explore the biologic and psychosocial basis for these results.

## Key Points

**Question:**

What are the rates of comorbidities and mortality among Holocaust survivors compared with control individuals?

**Findings:**

In this case-control study of 38 597 Holocaust survivors and 34 931 control individuals, significantly higher rates of comorbidities were reported in the Holocaust survivor group than in the control group. In contrast, the overall mortality rate was lower among Holocaust survivors (25.3%) compared with the control group (41.1%) after adjustment for confounders.

**Meaning:**

The findings suggest that although Holocaust survivors may experience more comorbidities, the mortality in the group may be lower, which may be associated with the improved health literacy and unique resilience characteristics among Holocaust survivors.

## Teaser

This case-control study uses nationwide health plan data from Israel to compare the extent of chronic comorbidities and mortality hazard between Holocaust survivors and an age-matched control group of individuals born in Israel.

## Introduction

Repeated studies have suggested that Holocaust survivors may experience more comorbidities, such as hypertension, heart diseases, osteoporosis, metabolic syndrome, and malignant diseases, compared with the general population.^1,2^ These consequences could be explained by psychosocial trauma and posttraumatic injury, poor hygienic conditions, prolonged malnutrition, and suboptimal preventive means.^3^ However, the overall survival and age at death among Holocaust survivors has been sparsely investigated.

The primary objective of this study was to quantify the mortality hazard among Holocaust survivors, compared with individuals from Israel born during the same years and participating in the same health care plan. In addition, we compared rates of comorbidities between Holocaust survivors and the control group.

## Methods

The study followed the Strengthening the Reporting of Observational Studies in Epidemiology (STROBE) reporting guideline.^4^ Data were collected from the computerized databases of Maccabi Healthcare Services, a nationwide health plan in Israel with more than 2 million members representing 25% of the population.^5^ Data are automatically collected and include information regarding all diagnoses, comorbidities, hospitalizations, emergency department visits, physician visits, outpatient specialist visits, and purchase of medications, laboratory test results, and radiologic imaging results. Each individual has a unique identification code in the system valid for all encounters. The database includes several automatically formulated disease registries, such as diabetes, hypertension, cardiovascular, cancer, and osteoporosis. Patients are identified by an automated database search, and the registry is not dependent on physicians actively reporting about the patient to the registry. These registries are routinely updated and validated by community physicians and other health care workers. Institutional review board approval and waiver of informed consent was obtained from Assuta hospital research ethics board, Tel Avia, Israel.

We located the Holocaust survivors by a special administrative mark of this population in the Maccabi Healthcare Service information systems and selected those who were born between 1911 (according to the oldest survivor) and 1945 in Europe. The control group included persons insured by Maccabi Healthcare Services who were born in Israel between the same years.

Socioeconomic status was categorized and ranged from 1 (the lowest) to 10 (the highest) based on the poverty index of the member’s enumeration area, as defined by Israel's 2008 national census.^6^ The poverty index is based on household income, education level, crowding, physical conditions, and car ownership. We verified the place of the child in the family and the total number of children in the family.

Two types of analyses were conducted. To assess the prevalence of chronic illness among members of both study groups, we checked for their presence in each of 11 chronic illness registries (including 5 types of heart disease [nonmyocardial infarction, myocardial infarction, cerebrovascular disease, congestive heart failure, and peripheral vascular disease], chronic kidney disease, chronic obstructive pulmonary disease, osteoporosis, diabetes, hypertension, and cancer) that Maccabi Healthcare Services maintains regularly and 2 additional medical conditions: dementia and obesity (body mass index [BMI] >30 [calculated as weight in kilograms divided by height in meters squared]). We examined the prevalence of these conditions in both study groups. This was a cross-sectional study using data collected from January 1, 1998, through December 31, 2017, with at least 2 previous years of membership in Maccabi Healthcare Services. In parallel, we compared the mortality hazard between the Holocaust survivors and the control group. The mortality data were available from 1988 until December 31, 2017.

### Statistical Analyses

Descriptive statistics are presented as number (percentage), mean (SD), or median (interquartile range). Comparisons of proportions used a χ^2^ test and means across time used a *t* test.

Logistic regression was conducted to compare the hazard of comorbidities between groups after adjustment for age, sex, socioeconomic status, and BMI. Cox regression and adjusted Kaplan-Meier curves were constructed to compare the age at death in each group with adjustment for sex, socioeconomic status, and BMI. All tests were 2 tailed with *P* < .05 defining statistical significance, and all analyses were conducted using SPSS, version 24 (SPSS Inc), standard statistical software.

## Results

### Cross-sectional Study

The Holocaust survivor group included 38 597 individuals, and the control group included 34 931 individuals, all of whom were present members of Maccabi Healthcare Services, Israel. The group of Holocaust survivors was significantly older (mean [SD] age, 81.7 [5.4] years vs 77.7 [5.3] years) and included more women (22 627 of 38 597 [58.6%] vs 18 615 of 34 931 [53.3%]) compared with the control group. Holocaust survivors also had a lower rate of high socioeconomic status (6216 [16.1%] vs 13 427 [38.4%]; *P* < .001). The mean (SD) number of comorbidities was higher in the survivor group (3.3 [1.7]) compared with the control group (2.7 [1.6]) (*P* < .001) (Table 1).

**Table 1.** Characteristics of Holocaust Survivor and Control Groups^a^

Characteristic	Holocaust Survivors (n = 38 597)	Control Group (n = 34 931)
Female	22 627 (58.6)	18 615 (53.3)
Age, mean (SD), y	81.7 (5.4)	77.7 (5.3)
Socioeconomic status^b^		
1-4	7805 (20.2)	5074 (14.5)
5-7	24 504 (63.5)	16 182 (46.3)
8-10	6216 (16.1)	13 427 (38.4)
Body mass index, mean (SD)^c^	28.4 (5.1)	27.6 (4.8)
Comorbidities, mean (SD)^d^	3.3 (1.7)	2.7 (1.6)

^a^ Data are presented as number (percentage) of individuals unless otherwise indicated. For all comparisons, *P* < .001.

^b^ Socioeconomic status of 1 to 4 was considered to be low and of 8 to 10 was considered to be high.

^c^ Calculated as weight in kilograms divided by height in meters squared.

^d^ Number of comorbidities ranged from 0 to 13.

The Holocaust survivors had higher rates than control individuals of reported hypertension (32 038 [83.0%] vs 23 285 [66.7]), obesity (12 838 [33.3%] vs 9254 [26.5]), chronic kidney disease (11 929 [30.9%] vs 6927 [19.8]), cancer (11 369 [29.5%] vs 9721 [27.8]), dementia (6389 [16.6%] vs 3355 [9.6]), ischemic heart disease, nonmyocardial infarction (5729 [14.8%] vs 4135 [11.8]), myocardial infarction (3641 [9.4%] vs 2723 [7.8]), and osteoporotic fractures among women (6429 [28.4%] vs 4120 [22.1]). By using logistic regression models to control for age, sex, BMI, and socioeconomic status, we found a higher morbidity hazard among survivors for hypertension (odds ratio [OR], 1.61; 95% CI, 1.54-1.67; *P* < .001), osteoporosis in women (OR, 1.11; 95% CI, 1.06-1.16; *P* < .001), obesity (OR, 1.36; 95% CI, 1.32-1.41; *P* < .001), chronic kidney disease (OR, 1.17; 95% CI, 1.13-1.22; *P* < .001), ischemic heart disease, nonmyocardial infarction (OR, 1.12; 95% CI, 1.07-1.18; *P* < .001), myocardial infarction (OR, 1.08; 95% CI, 1.02-1.14; *P* = .01), cerebrovascular disease (OR, 1.06; 95% CI, 1.01-1.11; *P* = .03), peripheral vascular disease (OR, 1.24; 95% CI, 1.15-1.34; *P* < .001), cancer (OR, 1.09; 95% CI, 1.05-1.13; *P* < .001), and dementia (OR, 1.08; 95% CI, 1.03-1.14; *P* = .002) (Table 2).

**Table 2.** Comparison of Comorbidity Rates Between Holocaust Survivor and Control Groups

Comorbidity	No. (%)		Odds Ratio (95% CI)^a^	*P* Value
	Holocaust Survivors (n = 38 597)	Control Group (n = 34 931)		
Hypertension	32 038 (83.0)	23 285 (66.7)	1.61 (1.54-1.67)	<.001
Osteoporosis				
Any	17 675 (45.8)	14 129 (40.4)	1.02 (0.98-1.05)	.43
Only women^b^	14 026 (62.0)	10 516 (56.5)	1.11 (1.06-1.16)	<.001
Fracture	8050 (20.9)	5676 (16.2)	1.00 (0.96-1.04)	.98
Only women^b^	6429 (28.4)	4120 (22.1)	1.06 (1.01-1.07)	.03
Diabetes	12 955 (33.6)	10 629 (30.4)	1.00 (0.97-1.04)	.99
Obesity, body mass index >30^c^	12 838 (33.3)	9254 (26.5)	1.36 (1.32-1.41)	<.001
Chronic kidney disease	11 929 (30.9)	6927 (19.8)	1.17 (1.13-1.22)	<.001
Cancer	11 369 (29.5)	9721 (27.8)	1.09 (1.05-1.13)	<.001
Dementia	6389 (16.6)	3355 (9.6)	1.08 (1.03-1.14)	.002
Ischemic heart disease, nonmyocardial infarction	5729 (14.8)	4135 (11.8)	1.12 (1.07-1.18)	<.001
Myocardial infarction	3641 (9.4)	2723 (7.8)	1.08 (1.02-1.14)	.01
Cerebrovascular disease	4772 (12.4)	3409 (9.8)	1.06 (1.01-1.11)	.03
Chronic obstructive pulmonary disease	3054 (7.9)	2809 (8.0)	0.98 (0.93-1.04)	.51
Congestive heart failure	2543 (6.6)	1480 (4.2)	1.02 (0.95-1.10)	.54
Peripheral vascular disease	2156 (5.6)	1417 (4.1)	1.24 (1.15-1.34)	<.001

^a^ The odds ratio was adjusted for age, sex, body mass index, and socioeconomic status using logistic regression.

^b^ Holocaust survivors (n = 22 627) and control group (n = 18 615).

^c^ Calculated as weight in kilograms divided by height in meters squared.

### Retrospective Cohort

The retrospective cohort section of the study included 51 644 patients (29 145 women [56.4%]) in the survivor group and 59 334 patients (30 168 women [50.8%]) in the control group who were born between 1911 and 1945 and had at least 2 previous years of membership in Maccabi Healthcare Services. When comparing mortality hazard rates between the 2 groups, 13 047 patients (25.3%) died in the Holocaust survivor group, which was significantly fewer than the number of deaths in the control group (24 403 [41.1%]) (*P* < .001). After adjustment for confounders, the mean (SD) age at death was 84.8 (6.6) years among the survivors compared with 77.7 (9.9) years among the controls (*P* < .001). The Cox regression showed that the difference in age at death between groups stayed higher in the survivor group compared with controls (hazard ratio, 0.47; 95% CI, 0.46-0.48; *P* < .001) after adjustment for sex, socioeconomic status, and BMI (Table 3 and the Figure).

**Table 3.** Cox Regression for Death Hazard Between Holocaust Survivor and Control Groups

Variable	Hazard Ratio (95% CI)^a^
Group	0.47 (0.46-0.48)
Sex	0.68 (0.67-0.69)
Body mass index	0.96 (0.958-0.959)
Socioeconomic status	0.93 (0.92-0.93)

^a^ All *P* < .001.

**Figure.**
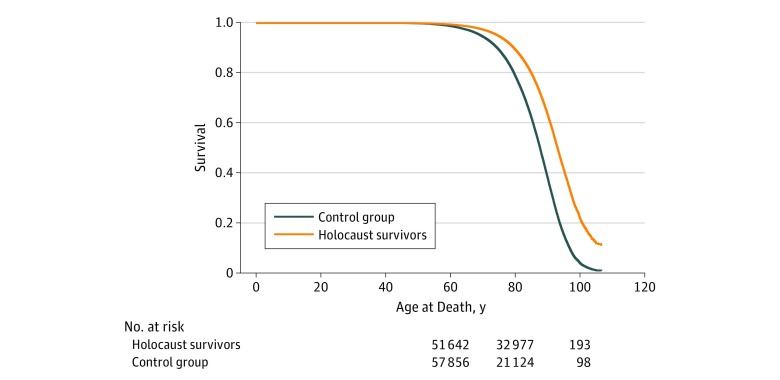
Adjusted Kaplan-Meier Curve for Mortality Hazard Between Holocaust Survivor and Control Groups

## Discussion

During the past decade, several studies^1,2^ have suggested that Holocaust survivors have higher rates of comorbidities compared with groups unexposed to genocide. Another study^7^ suggested an excess of psychosocial morbidities but not excessive physical comorbidities or changes in overall survival. However, the death hazard among Holocaust survivors has been only sparsely investigated or understood. Although Sagi-Schwartz and colleagues^8^ have suggested that exposure to the Holocaust was associated with a slight protective effect (ie, favorable longevity of approximately 6.5 months) mostly among men, Stessman and colleagues^7^ did not detect different rates of mortality.

The present study, to our knowledge, is the only study to date to compare comorbidities and mortality rates using the same cohort of survivors. Moreover, existing studies have not optimally controlled for confounders that may affect comorbidities or death hazard.

There is a broad understanding that a genocide experience sustained for 5 years would have serious consequences on the psychologic and physical well-being of individuals because of psychosocial trauma, posttraumatic injury, poor hygiene, prolonged malnutrition, and suboptimal preventive means.^3,8^ In the first part of our study, a cross-sectional analysis revealed excessive rates of comorbidities among Holocaust survivors after adjustment for age, sex, BMI, and socioeconomic status, with higher rates of hypertension, osteoporosis among women, diabetes, chronic renal disease, cancer, dementia, and cardiovascular illnesses.

However, higher rates of reported and diagnosed symptoms may theoretically reflect a potential reporting and ascertainment bias because Holocaust survivors may be more likely to seek medical help than matched controls owing to higher sensitivity to their health and better insight into health consequences. In addition, Holocaust survivors may be more likely to seek compensation and other types of privileges. However, our analysis revealed higher rates of comorbidities that were unlikely to be subject to ascertainment bias, such as confirmed myocardial infarction, cancer, and osteoporotic fractures. These results suggest that after adjusting for age, sex, socioeconomic class, and BMI, Holocaust survivors are more likely to develop serious comorbidities.

These results were contrary to our analysis of mortality hazard, in which Holocaust survivors had substantially greater survival compared with matched controls after controlling for potential confounders (Table 3). A potential reason for greater survival among Holocaust survivors may be associated with their death camp experience. Although many individuals in death camps died, those who survived may have had higher resilience from more favorable genetic, physical, and emotional characteristics.

There is ample evidence that serious morbidities, including cardiovascular morbidities and cancer, shorten life expectancy; thus, the contradiction of higher rates of comorbidities but lower death hazard among Holocaust survivors is interesting.^3^ This paradox may be explained in part by findings from a recent study by Elran-Barak et al^9^ that compared 164 Holocaust survivors with 134 prewar and 183 postwar immigrants to Israel regarding their coping strategies toward ensuring “the best possible life.” The study found that Holocaust survivors selected “maintaining good health” as a coping strategy almost twice as much as the other groups. Health literacy is defined as “the degree to which individuals have the capacity to obtain, process, and understand basic health information and services needed to make appropriate health decisions.”^10^ A study^11^ revealed that optimal health literacy may be associated with more favorable life expectancy because the individual is more likely to use preventive methods, participate in medical screening (eg, cancer and cardiovascular disease), and be diagnosed and treated earlier.

There may be other factors among Holocaust survivors that have not been appropriately quantified but that may be associated with improved ability to survive. It can be argued that the subgroup that survived the extreme conditions that many individuals did not survive had coping abilities that rendered them more resilient to comorbidities. For example, chronic stress has been shown to be associated with increased mortality risk among patients with atherosclerosis.^12^ It is conceivable that the stress response among Holocaust survivors is different so that these survivors are less sensitive to the consequences of some comorbidities. It has been shown that soldiers who have experienced combat-related trauma find greater meaning and satisfaction in their later lives secondary to these traumatic experiences.^3^

Resilience is commonly defined as adaptive characteristics of individuals to cope with and recover from adversity.^13^ Psychosocial determinants promoting resilience include optimism, cognitive flexibility, active coping skills, maintaining a supportive social network, attending to personal physical well-being, and embracing a personal moral compass.^14^ It is conceivable that the Darwinist ability to survive among Holocaust survivors who reached Israel was associated with favorable resilience despite the enduring consequences of serious comorbidities.

The present study may be important in understanding the favorable figures of life expectancy in Israel,^15^ because the genetic characteristics of Holocaust survivors may be associated with the long-term health of their children. Moreover, the study underscores the paucity of existing information about psychosocial determinants of longevity, such as health literacy and community support.

### Limitations

Potential weaknesses of the study need to be acknowledged. Date of death data were available from 1988 onward. In contrast, morbidity data were available from 1998 onward. We analyzed separate cohorts because we had no comorbidity information from 1988 to 1998 and we could not use it as a covariate. For comorbidity, we had to ensure that all participants were alive and that they had the opportunity to be diagnosed (thus we selected a cross-sectional analysis).

## Conclusions

Our study found higher rates of comorbidities and lower mortality among Holocaust survivors, which may be associated with improved health literacy and unique resilience characteristics among Holocaust survivors. More research is needed to explore the biologic and psychosocial basis for this resilience.

## References

[1] Keinan-Boker L, Shasha-Lavsky H, Eilat-Zanani S, Edri-Shur A, Shasha SM Chronic health conditions in Jewish Holocaust survivors born during World War II. Isr Med Assoc J. 2015;17(4):-. 26040044

[2] Bercovich E, Keinan-Boker L, Shasha SM Long-term health effects in adults born during the Holocaust. Isr Med Assoc J. 2014;16(4):203-207. 24834754

[3] Gallaway MS, Fink DS, Millikan AM, Mitchell MM, Bell MR The association between combat exposure and negative behavioral and psychiatric conditions. J Nerv Ment Dis. 2013;201(7):572-578. doi:10.1097/NMD.0b013e318298296a 23817154

[4] Equator Network. The Strengthening the Reporting of Observational Studies in Epidemiology (STROBE) Statement: guidelines for reporting observational studies. http://www.equator-network.org/reporting-guidelines/strobe. Accessed October 22, 2018.

[5] Shalev V, Chodick G, Goren I, Silber H, Kokia E, Heymann AD The use of an automated patient registry to manage and monitor cardiovascular conditions and related outcomes in a large health organization. Int J Cardiol. 2011;152(3):345-349. doi:10.1016/j.ijcard.2010.08.002 20826019

[6] Israel Central Bureau of Statistics Census of Population and Housing: Jerusalem 2008 http://www.cbs.gov.il/reader/cw_usr_view_Folder?ID=141. Accessed November 29, 2018.

[7] Stessman J, Cohen A, Hammerman-Rozenberg R, Holocaust survivors in old age: the Jerusalem Longitudinal Study. J Am Geriatr Soc. 2008;56(3):470-477. doi:10.1111/j.1532-5415.2007.01575.x 18194229

[8] Sagi-Schwartz A, Bakermans-Kranenburg MJ, Linn S, van Ijzendoorn MH Against all odds: genocidal trauma is associated with longer life-expectancy of the survivors. PLoS One. 2013;8(7):e69179. doi:10.1371/journal.pone.0069179 23894427 PMC3722177

[9] Elran-Barak R, Barak A, Lomranz J, Benyamini Y Proactive aging among Holocaust survivors: striving for the best possible life. J Gerontol B Psychol Sci Soc Sci. 2018;73(8):1446-1456. 27744368 10.1093/geronb/gbw136

[10] Centers for Disease Control and Prevention National Center for Health Statistics. Healthy People 2010 https://www.cdc.gov/nchs/healthy_people/hp2010.htm. Accessed on November 29, 2018.

[11] Flaherty JH Cancer screening in older patients: life expectancy, prioritization, and health literacy. Am Fam Physician. 2008;78(12):e186643. 19119549

[12] Meng LB, Qi R, Xu L, The more critical murderer of atherosclerosis than lipid metabolism: chronic stress. Lipids Health Dis. 2018;17(1):143. doi:10.1186/s12944-018-0795-4 29921279 PMC6009046

[13] Nugent NR, Sumner JA, Amstadter AB Resilience after trauma: from surviving to thriving. Eur J Psychotraumatol. 2014;5(1). doi:10.3402/ejpt.v5.25339 25317260 PMC4185140

[14] Iacoviello BM, Charney DS Psychosocial facets of resilience: implications for preventing posttrauma psychopathology, treating trauma survivors, and enhancing community resilience. Eur J Psychotraumatol. 2014;5(1). doi:10.3402/ejpt.v5.23970 25317258 PMC4185137

[15] The World: life expectancy (2018). http://www.geoba.se/population.php?pc=world&type=015&year=2018&st=country&asde=&page=1. Accessed October 23, 2018.

